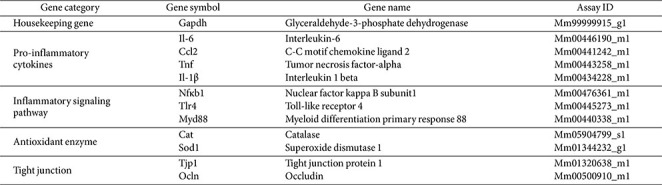# Heat-Killed *Lacticaseibacillus paracasei* HY2782 Attenuates Chronic Kidney Disease by Suppressing Inflammation and Selectively Modulating the Gut Microbial Community

**DOI:** 10.4014/jmb.2603.03043

**Published:** 2026-04-24

**Authors:** Hyeonji Kim, Ji-Woong Jeong, Joo-Yun Kim, Jae-Jung Shim, Jae-Hwan Lee

**Affiliations:** R & BD Center, hy Co. Ltd., 22, Giheungdanji-ro 24beon-gil, Giheung-gu, Yongin-si 17086, Republic of Korea

**Keywords:** Chronic kidney disease, Gut–kidney axis, Postbiotics, Gut microbiota, Inflammation

## Abstract

Chronic kidney disease (CKD) is a progressive disorder characterized by renal dysfunction, chronic inflammation, and gut microbial dysbiosis. In this study, we investigated the protective effect of heat-killed *Lacticaseibacillus paracasei* HY2782 (HY2782) in an adenine-induced CKD mouse model. Mice were evaluated for serum renal function markers, renal histopathology, inflammatory and barrier-related gene expression, and gut microbial composition. Oral administration of HY2782 significantly reduced serum blood urea nitrogen and creatinine levels and ameliorated renal histopathological damage, including tubular dilatation, cast formation, composite kidney injury, and mononuclear cell infiltration. In kidney tissue, HY2782 suppressed the CKD-associated upregulation of pro-inflammatory cytokine- and TLR4/NF-κB-related genes. In colon tissue, HY2782 attenuated the expression of pro-inflammatory cytokines and inflammatory signaling-related genes and showed a partial recovery trend in tight junction-related gene expression. Gut microbiota analysis revealed no significant changes in alpha diversity, whereas beta diversity was significantly altered by HY2782 treatment, indicating selective restructuring of the microbial community. At the taxonomic level, HY2782 significantly reduced *Mucispirillum* abundance and showed a recovery trend in *Akkermansia*, and correlation analysis further indicated that these taxa were associated with renal dysfunction and inflammatory markers. Collectively, these findings suggest that heat-killed HY2782 attenuates adenine-induced CKD in association with suppression of renal and intestinal inflammation and selective modulation of gut microbiota, supporting its potential as a postbiotic candidate for CKD management.

## Introduction

Chronic kidney disease (CKD) is a progressive and irreversible disorder characterized by a sustained decline in renal function and structural damage to kidney tissue. CKD affects an estimated 10–15% of the global population and is associated with a substantial increase in morbidity and mortality [1]. Pathological characteristics of CKD include impaired renal filtration capacity, renal fibrosis, tubular atrophy, increased albuminuria and/or proteinuria, and chronic inflammation, all of which contribute to disease progression and poor clinical outcomes [2,3]. CKD is categorized into five stages, ranging from mild renal impairment (stage 1) to end-stage renal disease (stage 5), based on the estimated glomerular filtration rate (eGFR) [4]. As CKD advances, renal function progressively declines, accompanied by metabolic disturbances, systemic inflammation, and an increased risk of cardiovascular events and mortality [5, 6].

Current therapeutic strategies for CKD primarily involve pharmacological treatments, such as renin–angiotensin–aldosterone system inhibitors, which focus on controlling blood pressure, blood glucose levels, and proteinuria [7]. Although such approaches can delay disease progression, complete prevention of the progressive decline in renal function remains unachieved. Consequently, many patients ultimately progress to end-stage renal disease, requiring dialysis or kidney transplantation. Therefore, alternative therapeutic strategies that improve CKD outcomes while minimizing adverse effects need to be developed [8, 9].

CKD is not simply a renal disorder but a systemic disease involving complex interactions between the kidney and other organs, particularly the gastrointestinal tract [10]. In particular, the gut–kidney axis has emerged as a novel therapeutic target in CKD, reflecting the close interaction between the intestinal environment and renal function [11]. In CKD, impaired renal filtration limits the excretion of uremic toxins, including indoxyl sulfate and p-cresyl sulfate, causing their accumulation in the circulation. These toxins induce oxidative stress and inflammatory responses in kidney tissue, thereby accelerating CKD progression [12]. In addition, increased intestinal permeability resulting from gut barrier dysfunction has been closely associated with CKD, allowing endotoxins such as lipopolysaccharide (LPS) to translocate into the circulation. These endotoxins trigger systemic inflammatory responses that exacerbate renal inflammation and exert additional stress on the kidney [13]. Consequently, chronic inflammation in CKD has been linked to disturbances in the gut environment, highlighting the contribution of the gut–kidney axis to disease progression.

Therefore, modulation of the gut environment has been proposed as a potential therapeutic approach for CKD [14]. Probiotics have been extensively studied for their beneficial effects on host health, such as regulating the gut–kidney axis, conferring immunomodulatory effects, and producing antimicrobial compounds [15,16]. However, the use of live probiotics requires careful consideration because of several limitations, including variability in product quality, limited shelf life, inconsistent efficacy, and challenges in administration to immunocompromised patients [17, 18]. Postbiotics, defined as non-viable microbial cells and/or their components that confer health benefits to the host, have emerged as safer alternatives to conventional probiotic interventions. Postbiotics exert physiological effects without requiring microbial viability [19, 20]. This characteristic may offer advantages in terms of safety and standardization, particularly in immunocompromised patients with CKD [21]. In addition, because heat-killed preparations do not depend on viability, they may offer practical advantages in terms of product stability, storage, and formulation consistency for translational applications.

Heat-killed *Lacticaseibacillus paracasei* HY2782 (formerly reported as *Lactobacillus casei* HY2782) reduces the mRNA expression of pro-inflammatory cytokines and restores the mRNA expression of tight junction-related genes in an LPS-induced Caco-2 cell model and a colitis animal model [22]. Although heat-killed HY2782 has demonstrated anti-inflammatory activity and modulatory effects on intestinal barrier-related functions, its potential role in ameliorating CKD in relation to gut–kidney axis-associated changes has not yet been elucidated.

Therefore, this study aimed to investigate the potential of heat-killed HY2782 as a novel postbiotic intervention for attenuating CKD progression. Using an adenine diet-induced CKD mouse model, we comprehensively evaluated physiological parameters and serum markers of renal function. Changes in renal histopathology and inflammation- and oxidative stress-related gene expression were analyzed. In parallel, intestinal inflammation, inflammatory signaling pathways, and tight junction-related gene expression were evaluated in colon tissue. Finally, we analyzed the intestinal microbiota and the correlations between phenotype markers and gut microbial taxa to explore the impact of microbial changes on CKD symptoms.

## Materials and Methods

### Preparation of Heat-Killed *Lacticaseibacillus paracasei* HY2782

*Lacticaseibacillus paracasei* HY2782 (formerly reported as *Lactobacillus casei* HY2782) was cultured in MRS broth (BD Difco, USA) at 37°C for 24 h under anaerobic conditions. After incubation, the bacterial cells were harvested by centrifugation at 4000 × *g* for 15 min. The cell pellets were washed twice and resuspended in phosphate-buffered saline and then heat treated at 100°C for 20 min. Complete inactivation was confirmed by the absence of colony formation on MRS agar after incubation. The resulting heat-killed *L. paracasei* HY2782 is referred to as “HY2782” in this study.

### Animal Experiments

**Fig. 1:**
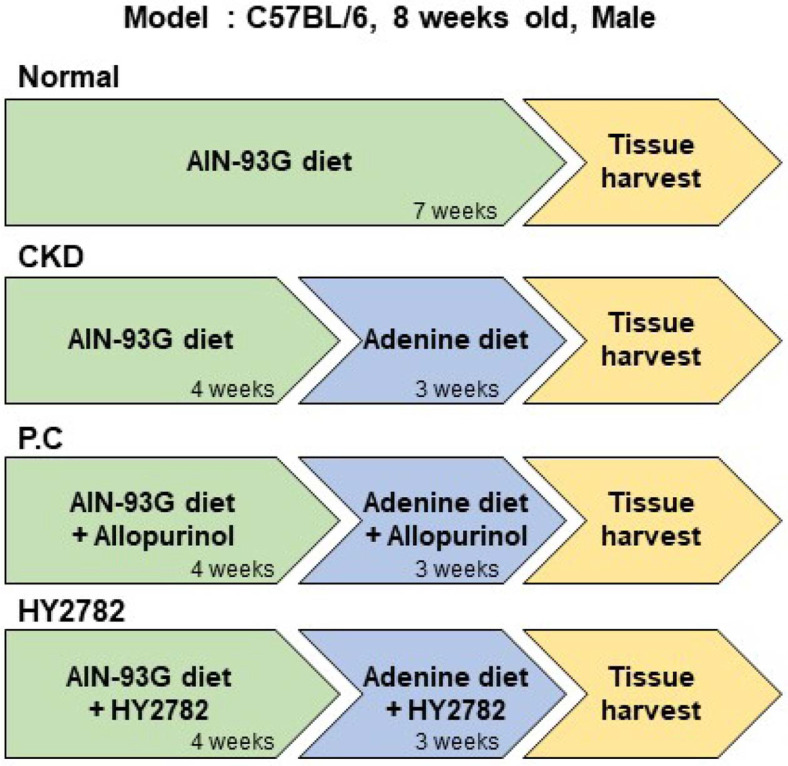
Flow chart of animal experiments.

Eight-week-old male C57BL/6 mice were obtained from Dooyeol Biotech (Republic of Korea) and acclimated in cages for 7 days under controlled conditions (50% ± 10% humidity, 23 ± 2°C, and a 12 h light/dark cycle). After the acclimation period, the mice were randomly assigned into four groups (*n* = 10 per group): normal group (Nor, fed an AIN-93G diet), CKD-induced group (CKD, fed a 0.15% adenine-containing AIN-93G diet), CKD-induced allopurinol (20 mg/kg/day)-treated group (P.C, positive control), and CKD-induced heat-killed HY2782 (10^9^ CFU/kg/day)-treated group (HY2782). Saline or the appropriate treatment was administered via oral gavage to each group. Four weeks after the initiation of sample administration, all mice, except those in the normal group, were fed a diet supplemented with 0.15% adenine for an additional 3 weeks to induce CKD. Body weight, water consumption, and food intake of mice were monitored weekly at the same time each day. At the end of the experiment, all mice were euthanized using a CO_2_ gas chamber. Blood, spleen, kidney, and colon tissues were harvested. Blood samples were centrifuged at 3000 × *g* for 15 min to separate the serum, and the spleen and kidney tissues were immediately weighed. The serum, kidney, and colon tissues were stored at -80°C until subsequent analyses. All animal procedures were approved by the Ethics Review Committee of the R&BD Center, hy Co., Ltd., Republic of Korea (approval number: AEC-2025-0003-Y). A flowchart outlining the experimental design is shown in Figure 1.

### Measurement of Kidney Function Indicator

Serum samples were separated and analyzed for blood urea nitrogen (BUN) and creatinine (Crea) levels using a 7180 Clinical Analyzer (Hitachi, Japan) at Dooyeol Biotech (Republic of Korea).

### Histopathological examination

Kidney tissue was carefully excised and promptly fixed in a 10% formalin solution. The fixed samples were embedded in paraffin, sectioned, and then stained with hematoxylin and eosin (H&E). The H&E-stained sections were captured using MoticDSAssistant (Motic VM V1 Viewer 2.0). Histological damage, including tubular dilatation, tubular necrosis, cast formation, and mononuclear cell infiltration, was evaluated in the H&E-stained sections. Injury was scored on a scale of 0–5 in non-overlapping visual fields at 400× magnification, with the following criteria: 0 (no injury), 1 (≤10%), 2 (11–25%), 3 (26–45%), 4 (46–75%) and 5 (≥75%). H&E staining and histological scoring were performed by experienced pathologists at Dooyeol Biotech (Seoul, Republic of Korea). All evaluations were conducted in a blinded manner to ensure objective and unbiased assessment.

### Gene Expression Analysis by Quantitative Real-Time PCR (qRT-PCR)

Total RNA was extracted from the kidney and colon tissues using the Easy-spin Total RNA Extraction Kit (iNtRON Biotechnology, Republic of Korea). The eluted RNA was reverse transcribed into complementary DNA (cDNA) at 37°C for 1 h using the Omniscript Reverse Transcription Kit (Qiagen, Germany). The concentration of the synthesized cDNA was quantified using an Agilent BioTek^®^ Synergy HT Microplate reader (Agilent Technologies, USA). Quantitative real-time PCR was conducted using the TaqMan^TM^ Gene Expression Master Mix (Applied Biosystems, USA) on a QuantStudio 6 Flex Real-time PCR system (Applied Biosystems). The genes analyzed in this study are listed in Table 1. The relative mRNA expression levels were normalized to those of glyceraldehyde-3-phosphate dehydrogenase (*Gapdh*; Mm99999915_g1).

### Preparation of Genomic DNA

For cecal tissue, seven randomly selected animals per group were used for analysis to ensure statistical representation. Total genomic DNA (gDNA) was extracted from mouse cecal samples using the QIAamp Fast DNA Stool Mini Kit (Qiagen) in accordance with the manufacturer’s instructions. The concentration of extracted gDNA was quantified using the Quant-iT™ PicoGreen^®^ dsDNA Assay Kit (Invitrogen, USA).

### 16S rRNA Gene Amplification, Library Preparation, and Sequencing

The V3–V4 regions of the bacterial 16S rRNA gene were amplified using the Illumina 16S metagenomic workflow. In brief, 5 ng of purified genomic DNA was subjected to PCR using Hercules II Fusion DNA Polymerase (Agilent Technologies) in the presence of reaction buffer, 1 mM dNTPs, and 500 nM of each primer. Thermal cycling was initiated with denaturation at 95°C for 3 min, followed by 25 amplification cycles of 95°C for 30 s, 55°C for 30 s, and 72°C for 30 s, and concluded with a final extension step at 72°C for 5 min.

Amplification employed primer sequences containing Illumina adapter overhangs: V3-F (5'-TCGTCGGCAGCGTCAGATGTGTATAA GAGACAGCCTACGGGNGGCWGCAG-3') and V4-R (5'-GTCTCGTGGGCTCGGAGATGTGTATAAGAGACAGGACTACHVGGGTAT CTAATCC-3').

PCR products were purified using AMPure XP beads (Beckman Coulter, USA). Index sequences were subsequently incorporated via a second PCR using the Nextera XT Index Kit (Illumina, USA), with amplification performed for 10 cycles under conditions identical to those described above. The indexed libraries were cleaned with magnetic beads and assessed for concentration and fragment size distribution using the KAPA Library Quantification Kit (KAPA Biosystems, USA) and Agilent D1000 ScreenTape system (Agilent Technologies, Germany). Sequencing was carried out in paired-end mode (2 × 300 bp) on the MiSeq platform (Illumina) at Macrogen (Republic of Korea).

### Bioinformatics Processing and Amplicon Sequence Variant Inference

Raw sequencing reads were processed using Cutadapt (v3.2) to remove residual adapters and primer sequences. Quality trimming was applied to truncate the forward and reverse reads to 250 and 200 bp, respectively. Amplicon sequence variants (ASVs) were inferred using the DADA2 pipeline (v1.18.0) implemented in R (v4.0.3). The workflow included quality filtering, error-rate learning, dereplication, denoising, and paired-end read merging. Reads with expected error values ≥2 were discarded prior to denoising. Chimeric sequences were identified and removed using the consensus-based removeBimeraDenovo function in DADA2. ASVs shorter than 350 bp were excluded from further analyses.

Normalization was performed using QIIME (v1.9.0) to account for variations in sequencing depth across samples. Rarefaction was conducted by subsampling each sample to the minimum sequencing depth observed across the dataset to ensure comparability. The resulting high-quality ASV table was used for subsequent ecological and taxonomic analyses.

### Taxonomic Assignment and Diversity Analysis

The taxonomic classification of ASVs was performed using a naïve Bayesian classifier implemented in DADA2 against the NCBI 16S reference database, with a confidence threshold of 50%. Downstream analyses were conducted using QIIME (v1.9.0). Alpha diversity was assessed using the observed ASVs, Chao1, and Faith’s phylogenetic diversity (PD) to evaluate within-sample microbial richness and phylogenetic complexity. Beta diversity was calculated based on Bray–Curtis dissimilarity. The microbial community structure was visualized using a principal coordinate analysis (PCoA) 2D plot. The statistical significance of variations in microbial community composition was evaluated using permutational multivariate analysis of variance (PERMANOVA), and pairwise PERMANOVA *p*-values were adjusted for multiple comparisons using the Benjamini–Hochberg procedure.

Sequencing data are available in the NCBI Sequence Read Archive under accession number PRJNA1441124.

### Correlation Heatmap

Spearman’s rank correlation analysis was performed to evaluate associations between microbial taxon abundances and host phenotypic parameters. Correlation coefficients (ρ) were calculated using normalized abundance data. The resulting correlation matrix was visualized as a heatmap using the ggplot2 package in R software (version 4.0.3). To account for multiple comparisons across all taxon–phenotype pairs, *p*-values were adjusted using the Benjamini–Hochberg false discovery rate (FDR) method. Statistical significance was defined as FDR-adjusted * *p* < 0.05, ** *p* < 0.01, and *** *p* < 0.001.

### Statistical Analysis

Data derived from the animal experiments, including physiological, biochemical, histological, and gene expression parameters, were analyzed and visualized using GraphPad Prism 10 software (GraphPad Software, USA). Results are expressed as the mean ± standard deviation (SD). Comparisons between groups were performed using one-way ANOVA followed by Tukey’s post hoc test. For microbial taxonomic analyses, group differences were evaluated using the Kruskal–Wallis test followed by Dunn’s post hoc test, and *p*-values were adjusted using the Benjamini–Hochberg FDR method. A *p*-value of less than 0.05 was considered statistically significant.

## Results

### Effects of HY2782 on Physiological Parameters and Serum Biochemical Markers in Adenine-Induced CKD Mice

**Fig. 2:**
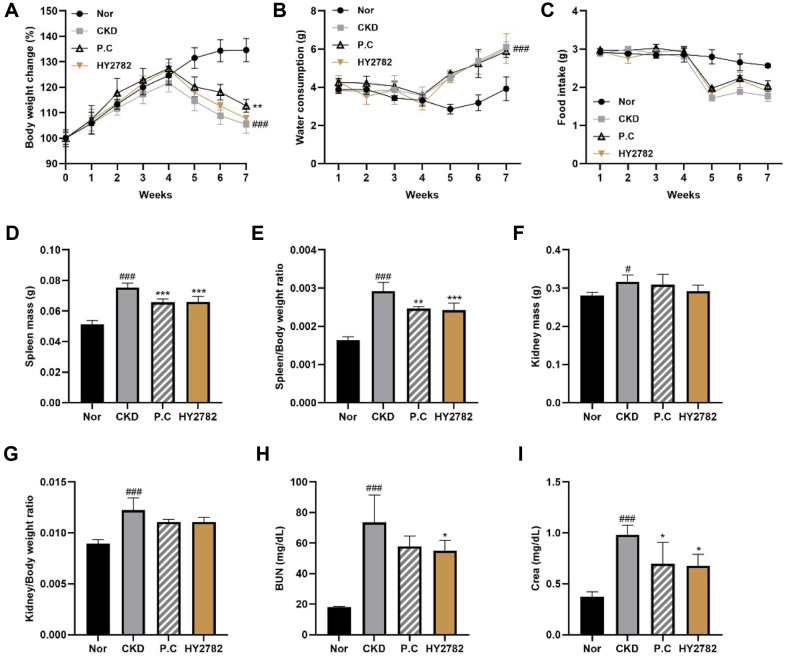
Effect of HY2782 on physiological and serum biochemical parameters of CKD-induced mice. (**A**) Changes in body weight, (**B**) water consumption, (**C**) food intake, (**D**) spleen mass, (**E**) spleen mass/body weight ratio, (**F**) kidney mass, (**G**) kidney mass/body weight ratio, (**H**) serum BUN, and (**I**) serum Crea. Results are presented as the mean ± standard deviation. ^#^*p* < 0.05 and ^###^*p* < 0.001 compared with the normal group. *p < 0.05, **p < 0.01, and ***p < 0.01 compared with the CKD group. Nor, normal group; CKD, adenine diet fed-CKD induced mice; P.C, adenine diet with positive control (allopurinol); HY2782, adenine diet with heat-killed *Lacticaseibacillus paracasei* HY2782; BUN, blood urea nitrogen; Crea, creatinine.

Changes in body weight, food and water intake, and spleen and kidney weights were assessed to comprehensively evaluate the effects of HY2782 on physiological and organ-related parameters in adenine diet-induced CKD mice (Fig. 2A–2G). The mice in the CKD group had significantly lower body weights than the mice in the normal group (*p* < 0.001). Treatment with P.C significantly attenuated body weight loss (*p* < 0.01). Treatment with HY2782 also reduced weight loss, but the difference was not statistically significant. Adenine diet-induced CKD also resulted in a significant increase in spleen weight and spleen/body weight ratio, both of which were significantly reduced following the administration of P.C or HY2782. In addition, kidney weight and kidney/body weight ratio were significantly higher in the CKD group than in the normal group (*p* < 0.05). Although treatment with P.C or HY2782 reduced these CKD-induced increases, the differences were not statistically significant compared with the CKD group.

The serum levels of biochemical markers BUN and Creatinine (Crea) were measured to further assess renal function in mice with adenine diet-induced CKD. As shown in Fig. 2H and 2I, the serum levels of BUN and Crea were significantly higher in the CKD group than in the normal group (*p* < 0.001). Treatment with HY2782 significantly downregulated the levels of BUN and Crea compared with those in the CKD group (*p* < 0.05). Treatment with P.C also reduced the levels of BUN and Crea, but statistical significance was only reached for Crea (*p* < 0.05). Notably, treatment with HY2782 showed a more consistent improvement in serum renal function markers than treatment with P.C.

### Effects of HY2782 on Renal Histopathological Alterations in Adenine-Induced CKD Mice

**Fig. 3:**
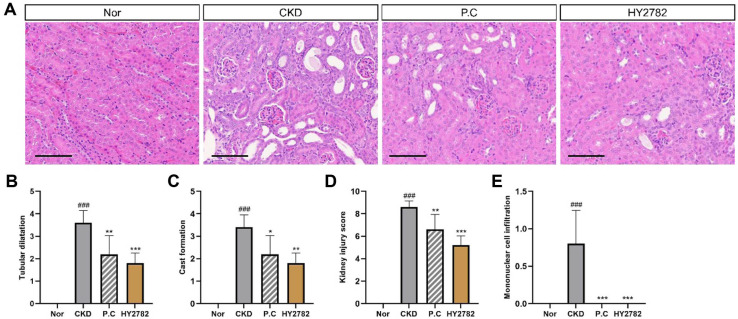
Histopathological analysis of kidney tissue in CKD-induced mice. (**A**) Images of H&E stained-sections (400× magnification, Scale bar = 100 μm. Scores of (**B**) tubular dilatation, (**C**) cast formation, (**D**) kidney injury, and (**E**) mononuclear cell infiltration. Results are presented as the mean ± standard deviation. ^###^*p* < 0.001 compared with the normal group. *p < 0.05, **p < 0.01, and ***p < 0.01 compared with the CKD group. Nor, normal group; CKD, adenine diet fed-CKD induced mice; P.C, adenine diet with positive control (allopurinol); HY2782, adenine diet with heat-killed *Lacticaseibacillus paracasei* HY2782; BUN, blood urea nitrogen; Crea, creatinine.

The kidney tissue was subjected to H&E staining and assessed for injury to evaluate renal damage. The H&E-stained kidney sections from the normal mice displayed unremarkable renal morphology, whereas those from the adenine-induced CKD mice exhibited abnormal renal histological features characterized by tubular necrosis, tubular dilatation, and cast formation (Fig. 3A). Treatment with either P.C or HY2782 alleviated these histopathological changes. As illustrated in Fig. 3B and 3C, the scores for tubular dilatation and cast formation were significantly higher in the CKD group than in the normal group (*p* < 0.001). By contrast, the score for tubular necrosis remained constant at 1.00 across the CKD, P.C, and HY2782 groups. As shown in Figure 3D, the score for composite kidney injury, calculated by combining tubular dilatation, cast formation, and tubular necrosis, was significantly higher in the CKD group than in the normal group (*p* < 0.001). Treatment with either P.C (*p* < 0.01) or HY2782 (*p* < 0.001) significantly attenuated the increase in kidney injury score, with a numerical reduction observed in the HY2782 group. In addition, the mice in the CKD group exhibited a pronounced increase in mononuclear cell infiltration (*p* < 0.001), which was effectively mitigated by administration of P.C or HY2782 (*p* < 0.001).

### Effects of HY2782 on the Expression of Genes Encoding Inflammatory Cytokines and Antioxidant Enzymes in Kidney Tissue

The mRNA expression of pro-inflammatory cytokines and NFκB/TLR signaling pathway-related genes was significantly upregulated in the CKD group (*p* < 0.001). In specific, the relative mRNA levels of *Il-6*, *Ccl2*, and *Nfκb1* increased 92.58-, 105.04-, and 6.12-fold, respectively, in the CKD group compared with the normal group (1.00-fold). Administration of HY2782 markedly attenuated the CKD-induced upregulation of *Il-6*, *Ccl2*, and *Nfκb1* by 61.59-fold (*p* < 0.05), 82.56-fold (not significant), and 4.51-fold (*p* < 0.05), respectively. The P.C group showed a modest reduction, but the magnitude of reduction appeared smaller than that observed in the HY2782 group. The adenine diet significantly increased the mRNA level of *Tlr4* (*p* < 0.001), whereas treatment with P.C and HY2782 significantly alleviated this upregulation (*p* < 0.05).

**Fig. 4:**
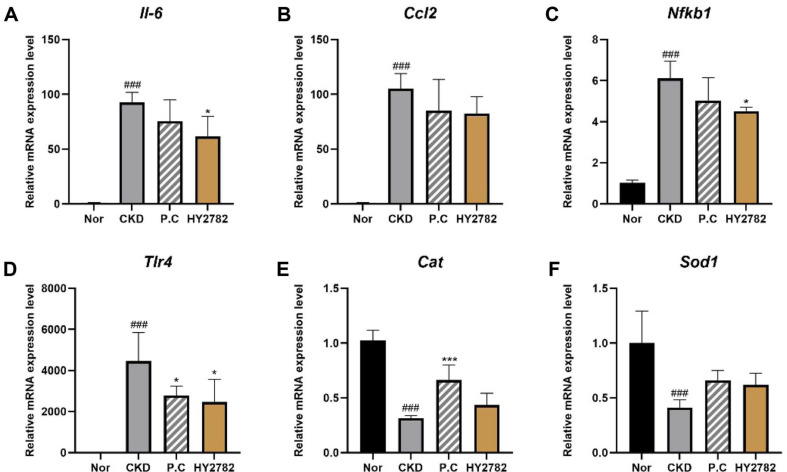
Effect of HY2782 on gene expression in kidney tissue. Relative expression of mRNA encoding pro-inflammatory cytokines (**A**) *Il-6* and (**B**) *Ccl2*, relative expression of mRNA encoding inflammatory signaling pathway (**C**) *Nfκb1* and (**D**) *Tlr4*, and relative expression of mRNA encoding antioxidant enzyme (**E**) *Cat* and (**F**) *Sod1*. Results are presented as the mean ± standard deviation. ^###^ *p* < 0.001 compared with the normal group. *p < 0.05 and ***p < 0.01 compared with the CKD group. Nor, normal group; CKD, adenine diet fed-CKD induced mice; P.C, adenine diet with positive control (allopurinol); HY2782, adenine diet with heatkilled *Lacticaseibacillus paracasei* HY2782; *Il-6*, interleukin-6; *Ccl2*, C-C motif chemokine ligand 2; *Nfκb1*, nuclear factor kappa B subunit 1; *Tlr4*, toll-like receptor 4; *Cat*, catalase; *Sod1*, superoxide dismutase 1.

We assessed the mRNA expression levels of genes encoding antioxidant enzymes, including *Cat* and *Sod1*. As shown in Fig. 4E–4F, the mRNA levels of *Cat* and *Sod1* significantly decreased 0.32- and 0.41-fold, respectively, in the CKD group compared with the normal group (*p* < 0.001). The mRNA levels of *Cat* and *Sod1* increased 0.44- and 0.62-fold, respectively, in the HY2782 group compared with the CKD group, but the differences were not statistically significant. In the P.C group, the mRNA expression levels of *Cat* and *Sod1* increased 0.66- and 0.65-fold, respectively, but only the increase in *Cat* expression was statistically significant (*p* < 0.001).

### Effects of HY2782 on the Expression of Genes Encoding Inflammatory and Tight Junction in Colon Tissue

**Fig. 5:**
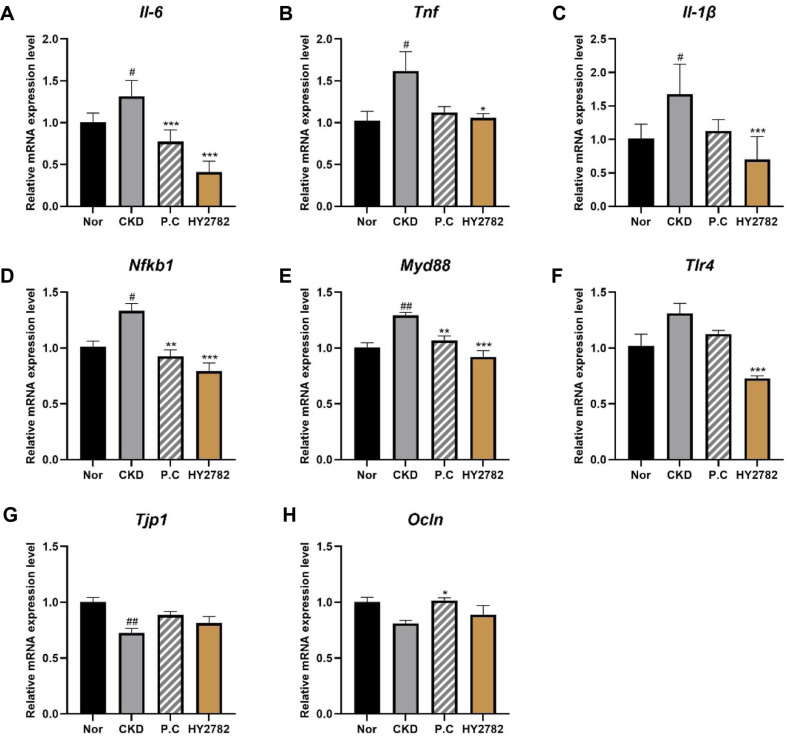
Effect of HY2782 on gene expression in colon tissues. Relative mRNA expression of pro-inflammatory cytokines (**A**) *Il-6*, (**B**) Tnf, and (**C**) *Il-1β*; inflammatory signaling pathway-related genes (**D**) *Nfκb1*, (**E**) *Myd88*, and (**F**) *Tlr4*; and tight junction-related genes (**G**) *Tjp1* and (**H**) *Ocln*. Results are presented as the mean ± standard deviation. ^#^*p* < 0.05 and ^##^*p* < 0.01 compared with the normal group. *p < 0.05, **p < 0.01, and ***p < 0.01 compared with the CKD group. Nor, Normal group; CKD, adenine diet fed-CKD induced mice; P.C, adenine diet with positive control (allopurinol); HY2782, adenine diet with heat-killed *Lacticaseibacillus paracasei* HY2782; *Il-6*, interleukin-6; *Tnf*, tumor necrosis factor alpha; *Il-1β*, interleukin-1 beta; *Nfκb1*, nuclear factor kappa B subunit 1; *Myd88*, myeloid differentiation primary response 88; *Tlr4*, toll-like receptor 4; *Tjp1*, tight junction protein 1; *Ocln*, occludin 1.

To explore whether adenine-induced CKD is associated with intestinal inflammatory responses, we evaluated the expression levels of genes related to pro-inflammatory cytokines and inflammatory signaling pathways in colon tissue (Fig. 5). The mRNA expression levels of the pro-inflammatory cytokines *Il-6*, *Tnf*, and *Il-1β* significantly increased 1.32-, 1.62-, and 1.68-fold, respectively, in the CKD group compared with the normal group (*p* < 0.05). As shown in Figure 5, treatment with HY2782 significantly attenuated the CKD-induced upregulation of these cytokines by 0.41-fold (*Il-6*, *p* < 0.001), 1.06-fold (*Tnf*, *p* < 0.05), and 0.70-fold (*Il-1β*, *p* < 0.001), respectively. Treatment with P.C also reduced the expression levels of these cytokines; however, only the decrease in *Il-6* expression was statistically significant (*p* < 0.001). These findings prompted further analysis of the upstream inflammatory signaling pathways associated with intestinal inflammation in CKD.

To determine whether the alterations in pro-inflammatory cytokine expression were accompanied by changes in inflammatory signaling pathways, we next examined the mRNA expression levels of genes involved in the NF-κB/TLR signaling pathway in colon tissue. The mRNA levels of *Nfκb1* and *Myd88* increased 1.33-fold (*p* < 0.05) and 1.29-fold (*p* < 0.01), respectively, in the CKD group compared with the normal group. The expression of these genes significantly decreased in the P.C and HY2782 groups compared with the CKD group, with HY2782 treatment significantly reducing *Nfκb1* and *Myd88* expression by 0.79- and 0.92-fold, respectively (*p* < 0.001). The mRNA expression level of *Tlr4* in colon tissue increased 1.31-fold in the CKD-induced group compared with the normal group, but the difference was not statistically significant. *Tlr4* expression decreased 1.13-fold in the P.C group compared with the CKD group, but the difference was not statistically significant. By contrast, *Tlr4* expression significantly decreased 0.73-fold in the HY2782 group compared with the CKD group (*p* < 0.001).

Taken together, these results indicate that administration of HY2782 consistently attenuates CKD-induced intestinal inflammation by reducing the expression of pro-inflammatory cytokines and NF-κB/TLR signaling pathway-related genes.

To determine whether HY2782 affects intestinal barrier integrity in adenine diet-induced CKD mice, we examined the mRNA expression levels of tight junction-related genes in colon tissue. The expression levels of *Tjp1* and *Ocln* decreased 0.73-fold (*p* < 0.01) and 0.81-fold (not significant), respectively, in the CKD group compared with the normal group. By contrast, these levels increased in the P.C or HY2782 group compared with the CKD group.

### Effect of HY2782 on the Gut Microbiota of Mice with Adenine Diet-Induced CKD

**Fig. 6:**
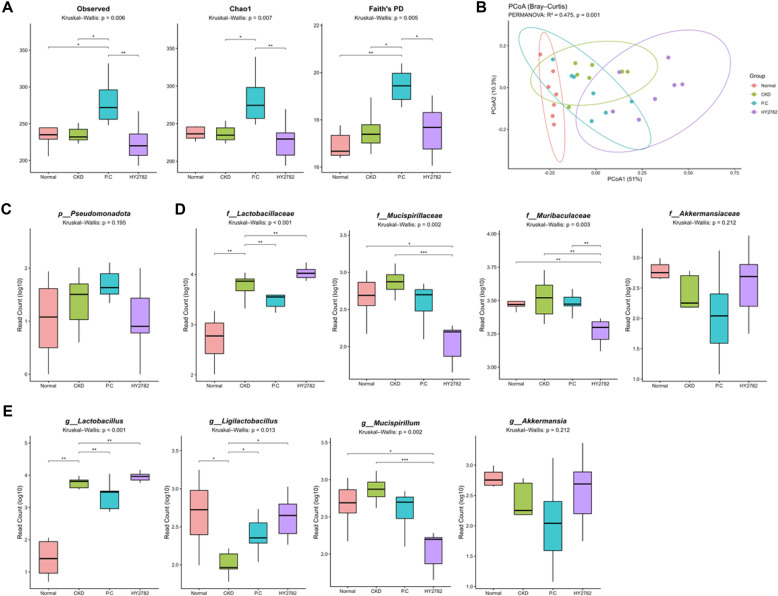
Composition of the gut microbiota in the adenine diet-induced CKD mice (*n* = 7/group). (**A**) α-diversity, (**B**) β-diversity, (**C**) taxonomy abundance at the phylum, (**D**) family and (**E**) genus level. Red box represents the normal group, green box represents the CKD group, blue box represents the P.C group, and purple box represents the HY2782 group. Bold black lines mean the median. Group differences were analyzed using the Kruskal–Wallis test followed by Dunn’s post hoc test with Benjamini–Hochberg FDR correction. Adjusted *p*-values were used to determine statistical significance (*p < 0.05, ** *p* < 0.01, *** *p* < 0.001). The overall Kruskal–Wallis *p*-value is displayed in the subtitle, and significant pairwise comparisons are indicated by asterisks. CKD, adenine diet fed-CKD induced mice; P.C, adenine diet with positive control (allopurinol); HY2782, adenine diet with heat-killed *Lacticaseibacillus paracasei* HY2782.

To investigate whether HY2782 supplementation modulates gut microbial composition in adenine diet-induced CKD mice, we performed 16S rRNA gene sequencing on cecum samples collected from each experimental group (Fig. 6). Alpha diversity was evaluated using observed features, Chao1, and Faith’s PD. No significant differences in these parameters were observed between the normal and CKD groups, indicating that adenine-induced CKD did not markedly affect overall microbial richness or phylogenetic diversity. Similarly, HY2782 supplementation did not significantly alter alpha diversity. By contrast, treatment with P.C significantly affected alpha diversity indices, indicating an intervention-associated shift in the overall microbial diversity.

Beta diversity was assessed to determine whether the overall microbial community structure differs among the groups. Groupwise differences in community composition were visualized through Bray–Curtis dissimilarity-based PCoA. PERMANOVA results indicated a significant difference in the overall microbial community structure across groups (R^2^ = 0.475, *p* = 0.001). Pairwise comparisons revealed significant differences between the normal and CKD groups (*p* = 0.003), consistent with CKD-associated compositional alterations. The HY2782 group also differed significantly from the CKD group (*p* = 0.003). By contrast, the P.C group did not differ significantly from the CKD group (*p* = 0.06). Moreover, the microbial community profiles differed significantly between the HY2782 and P.C groups (*p* = 0.002).

To further delineate the taxonomic alterations underlying the observed beta diversity differences, we evaluated microbial composition at the phylum, family, and genus levels using log10-transformed read counts. At the phylum level, Pseudomonadota (formerly Proteobacteria) was more abundant in the CKD group than in the normal group, but the difference was not statistically significant. HY2782 supplementation showed a non-significant decreasing trend relative to the CKD group. At the family level, *Lactobacillaceae*, *Mucispirillaceae*, and *Muribaculaceae* were more abundant in the CKD group than in the normal group, whereas *Akkermansiaceae* abundance was reduced. *Mucispirillaceae* and *Muribaculaceae* were less abundant in the HY2782 group than in the CKD group. Meanwhile, the abundances of *Lactobacillaceae* and *Akkermansiaceae* increased in the HY2782 group; however, these differences were not statistically significant. At the genus level, the abundances of *Lactobacillus* and *Mucispirillum* increased and those of *Ligilactobacillus* and *Akkermansia* decreased in the CKD group compared with the normal group. The abundance of *Lactobacillus* further increased in the HY2782 group compared with the CKD group. In addition, the abundances of *Ligilactobacillus* and *Akkermansia* were elevated in the HY2782 group but did not differ significantly from those observed in the normal group. Notably, *Mucispirillum* abundance was significantly lower in the HY2782 group than in the CKD group. These genus-level patterns were concordant with family-level changes, consistent with the taxonomic relationships among *Akkermansia*/*Akkermansiaceae*, *Mucispirillum*/*Mucispirillaceae*, and *Lactobacillus*/*Lactobacillaceae*.

### Correlation Analysis

**Fig. 7:**
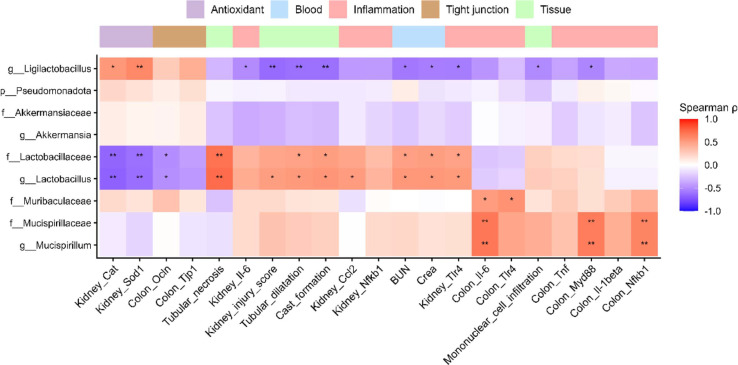
Spearman correlation heatmap between host physiological parameters and gut microbial taxa. Positive correlations are indicated in red and negative correlations in blue, with color intensity proportional to the strength of the association. *p*-values were adjusted for multiple comparisons using the Benjamini–Hochberg false discovery rate method. Statistical significance is indicated as follows: *p < 0.05, ** *p* < 0.01, *** *p* < 0.001. Phenotypic parameters were grouped into functional categories including blood, inflammation, antioxidant, tight junction, and tissue-related markers, which are displayed as an annotation bar above the heatmap.

To further investigate potential associations between altered microbial taxa and host physiological parameters, we performed Spearman’s correlation analysis between taxon abundance and renal and physiological parameters (Fig. 7). The abundances of *Akkermansia* and *Akkermansiaceae* negatively correlated with the levels of renal dysfunction markers, including serum BUN and Crea, and renal inflammatory markers. By contrast, the abundances of *Mucispirillum* and *Mucispirillaceae* significantly positively correlated with the levels of colonic inflammatory cytokines, including *Il-6*, *Il-1β*, and *Nfκb1*, as well as mononuclear cell infiltration scores. Interestingly, *Lactobacillus* and *Lactobacillaceae* significantly positively correlated with renal injury markers and inflammatory indices, including serum BUN, Crea, and selected pro-inflammatory cytokines. Statistically significant correlations are indicated with asterisks on the heat map. Although these correlations do not establish causality, they indicate potential associations between specific microbial taxa and host pathophysiological parameters in adenine-induced CKD. These associations were examined to identify potential host–microbe relationships and should therefore be regarded as exploratory rather than causal.

## Discussion

In the present study, we demonstrated that heat-killed HY2782 alleviates adenine-induced CKD by improving renal function, attenuating renal histopathological injury, and modulating inflammation- and oxidative stress-related responses. Importantly, these renoprotective effects were accompanied by the suppression of intestinal inflammation and gut-derived inflammatory signaling, supporting the potential role of the gut–kidney axis in mediating the beneficial effects of HY2782.

An adenine-induced CKD mouse model was used to evaluate the effects of HY2782 on CKD progression. This model is widely used in CKD research because it reliably induces renal dysfunction and mimics the key features of human CKD [23]. Adenine administration leads to the accumulation of 2,8-dihydroxyadenine (2,8-DHA), resulting in kidney damage, including impaired renal function, renal tubular injury, and increased serum BUN and Crea levels. Furthermore, the accumulation of 2,8-DHA crystals in the renal tubules leads to obstruction, which not only directly affects the tubules but also exerts pressure on the peritubular capillaries, ultimately leading to renal inflammation and oxidative stress [24-26].

Administration of an adenine diet induces various alterations, including body weight loss, elevated water consumption, reduced food intake, and spleen and kidney enlargement. The body weight loss in adenine-fed CKD mice is generally attributed to uremia-associated anorexia, metabolic acidosis, and chronic inflammation, which collectively impair nutrient utilization and energy homeostasis [27-29]. Moreover, renal dysfunction promotes the accumulation of uremic toxins, which may disrupt appetite regulation and promote a catabolic state [30]. The increase in water intake likely reflects an impaired renal concentrating capacity secondary to tubular injury. Intratubular crystal deposition and interstitial damage compromise tubular reabsorption, leading to increased urine output and compensatory polydipsia. These disturbances in fluid homeostasis are characteristic of adenine-induced CKD [31-33]. In addition to these metabolic and fluid disturbances, an increase in spleen weight may indicate systemic inflammation and immune activation accompanying CKD progression [34]. Notably, HY2782 supplementation improved physiological parameters comparable to P.C treatment. Serum BUN and Crea levels significantly increased in the adenine-induced CKD mice. As established markers of renal function, these elevations indicate compromised glomerular filtration capacity [35]. HY2782 administration significantly reduced BUN and Crea levels, indicating an improvement in renal functional capacity.

Consistent with the improvement in serum renal markers, histopathological examination of kidney tissue further demonstrated the protective effects of HY2782. Histopathological assessment revealed marked tubular dilatation, cast formation, and tubular necrosis in the adenine-induced CKD mice. Tubular dilatation may reflect impaired tubular integrity and increased intraluminal pressure secondary to crystal-induced obstruction. Cast formation is generally considered indicative of the accumulation of proteinaceous debris and cellular remnants within injured tubules, suggesting the disruption of tubular reabsorptive function. Tubular necrosis indicates severe epithelial damage, which may be driven by sustained crystal deposition, local inflammation, or hypoxic stress. Collectively, these structural alterations are characteristic of adenine-induced renal injury and contribute to progressive renal dysfunction [23,36,37]. Renal injury scores were numerically lower in the HY2782 group than in the P.C group, although the difference between the two treatment groups was not statistically significant.

In addition to attenuating structural damage, HY2782 suppressed renal inflammatory responses in the adenine-induced CKD mice. The mRNA expression levels of pro-inflammatory cytokines, including *Tnf* and *Il-6*, as well as inflammatory signaling-related genes, such as *Nfκb1* and *Tlr4*, were elevated in the CKD group but reduced in the HY2782 group. Both HY2782 and P.C decreased the expression of inflammatory genes, but HY2782 tended to show greater downregulation for some markers. The reduction in mononuclear cell infiltration further supported the involvement of HY2782 in the regulation of renal inflammatory pathways in this model.

Given the close interplay between inflammation and oxidative stress in CKD progression, antioxidant defense-related gene expression was further evaluated in kidney tissue [38]. Oxidative stress is a key contributor to CKD progression, and the mRNA expression levels of antioxidant enzymes *Cat* and *Sod1* significantly reduced in the adenine-induced CKD mice. HY2782 administration resulted in a modest increase in the expression of these genes; however, these changes were not statistically significant. By contrast, P.C treatment exhibited a relatively greater enhancement of antioxidant gene expression. Collectively, these findings suggest that the renoprotective effects of HY2782 in this model are mediated primarily through anti-inflammatory mechanisms, with limited involvement of direct upregulation of antioxidant gene expression.

CKD is increasingly recognized as a systemic disorder characterized by complex inter-organ crosstalk, with renal inflammation closely associated with intestinal immune dysregulation [39]. The gut–kidney axis represents a key pathway through which intestinal inflammation and barrier dysfunction may accelerate CKD progression [40]. In this context, inflammatory signaling in the colon tissue was further evaluated to determine whether HY2782 exerts broader regulatory effects along this axis.

Consistent with the concept of gut–kidney crosstalk in CKD, adenine-induced CKD mice exhibited a clear pro-inflammatory profile in colon tissue. The expression levels of the pro-inflammatory cytokines *Tnf*, *Il-6*, and *Il-1β* were elevated in the CKD group, together with upregulation of key inflammatory signaling genes, including *Nfκb1*, *Myd88*, and *Tlr4*. Importantly, HY2782 intake significantly attenuated these CKD-associated increases, indicating that intestinal inflammatory signaling was suppressed in parallel with the improvement of renal inflammatory parameters. P.C also reduced the expression of colonic inflammatory genes, but HY2782 tended to show greater downregulation for several markers. These findings suggest that HY2782 modulates intestinal immune responses under CKD conditions and that such changes are consistent with a role of gut–kidney axis-related regulation. The present study did not directly evaluate functional mediators such as microbial metabolites, circulating endotoxin, or intestinal permeability. Therefore, the mechanistic links between intestinal changes and renal protection remain to be clarified.

Intestinal inflammation is closely linked to impaired epithelial barrier integrity in CKD [41]. Sustained inflammatory signaling can disrupt tight junction structures, thereby increasing intestinal permeability and facilitating systemic exposure to gut-derived endotoxins [42]. To explore whether HY2782 influences barrier-related alterations, the expression of tight junction-associated genes was evaluated in colon tissue. The mRNA levels of *Tjp1* and *Ocln*, which are essential components of the epithelial barrier, were reduced in the adenine-induced CKD mice, consistent with compromised barrier integrity [43]. HY2782 treatment resulted in partial restoration of these transcripts; however, the observed changes were not statistically significant. HY2782 clearly reduced intestinal inflammatory signaling, whereas the restoration of tight junction gene expression was less robust. This pattern suggests that barrier recovery may require prolonged intervention or additional regulatory mechanisms.

Intestinal inflammation and barrier dysfunction are closely associated with microbial dysbiosis in CKD [44]. Accordingly, we examined whether HY2782 administration modulates gut microbiota composition. In the present study, adenine-induced CKD did not result in significant alterations in global microbial richness or phylogenetic diversity, as indicated by stable alpha diversity indices. This finding suggests that CKD-associated dysbiosis in this model is not characterized by a generalized loss of microbial diversity but rather by the compositional restructuring of specific taxa. Such patterns are increasingly recognized in chronic inflammatory conditions, where shifts in community structure may occur without substantial changes in overall richness.

Consistent with this interpretation, beta diversity analysis revealed significant differences in microbial community composition among the groups. The community structure in the HY2782 group was distinct from that in the CKD group despite the absence of alpha diversity changes. These findings indicate that HY2782 selectively modulates microbial composition rather than broadly enhancing microbial diversity.

Taxonomic analyses revealed hierarchical alterations at the family and genus levels. In this model, CKD was associated with enrichment of *Mucispirillaceae*/*Mucispirillum* and depletion of *Akkermansiaceae*/*Akkermansia*, taxa previously reported to be associated with intestinal inflammation, barrier dysfunction, and CKD-related dysbiosis [45,46]. HY2782 supplementation reduced *Mucispirillum* abundance and showed a recovery trend in *Akkermansia*, suggesting selective modulation of microbial taxa associated with intestinal inflammatory status. These patterns may be biologically relevant in the context of CKD, because *Mucispirillum* has been linked to inflammatory conditions, whereas *Akkermansia* has been associated with mucosal homeostasis and barrier-related functions. Several microbial changes were modest and did not reach statistical significance. In addition, genus-level abundance does not necessarily reflect strain-level function. These findings should therefore be interpreted cautiously. Thus, the observed taxonomic shifts are more appropriately viewed as selective ecological changes associated with HY2782 treatment rather than direct evidence of causal microbial mechanisms.

Interestingly, *Lactobacillus*-related taxa positively correlated with renal injury and inflammatory markers in this model. *Lactobacillus* is often regarded as beneficial, but its biological significance at the genus level may vary depending on host condition, strain composition, and the overall microbial context. Therefore, the observed correlations should not be interpreted as direct mechanistic evidence but rather as exploratory associations within the microbial restructuring induced under CKD conditions. The further increase observed following HY2782 supplementation may reflect context-dependent ecological reshaping rather than a straightforward protective expansion.

Taken together, the present findings indicate that heat-killed HY2782 attenuates adenine-induced CKD progression in association with suppression of renal inflammation, attenuation of intestinal inflammatory signaling, and selective modulation of the gut microbial community. These results are consistent with potential regulation along the gut–kidney axis. However, the current study does not establish a direct causal mechanism linking microbial alterations to renal protection.

Some limitations should be considered. First, the study was conducted using a single animal model of CKD and included only male mice, which may limit the generalizability of the findings. Second, although associations between microbial taxa and host parameters were identified, the study did not directly evaluate microbial metabolites, uremic toxins, or functional intestinal permeability. Third, some microbiota and barrier-related changes were modest, and therefore the corresponding interpretations should remain cautious. Future studies addressing these factors will be necessary to further clarify the mechanisms underlying HY2782-mediated renoprotection.

## Figures and Tables

**Table 1:** TaqMan probes used in in vivo experiments.